# ^18^F-FDG PET/CT radiomics for prediction of lymphovascular invasion in patients with early stage non-small cell lung cancer

**DOI:** 10.3389/fonc.2023.1185808

**Published:** 2023-07-21

**Authors:** Jie Wang, Zhonghang Zheng, Yi Zhang, Weiyue Tan, Jing Li, Ligang Xing, Xiaorong Sun

**Affiliations:** ^1^ Department of Graduate, Shandong First Medical University and Shandong Academy of Medical Sciences, Jinan, Shandong, China; ^2^ Department of Nuclear Medicine, Shandong Cancer Hospital and Institute, Shandong First Medical University and Shandong Academy of Medical Sciences, Jinan, Shandong, China; ^3^ Department of Radiation Oncology, Shandong Cancer Hospital and Institute, Shandong First Medical University and Shandong Academy of Medical Sciences, Jinan, Shandong, China

**Keywords:** lymphovascular invasion, lung cancer, positron emission tomography computed tomography, radiomics, nomogram

## Abstract

**Objective:**

To explore a prediction model for lymphovascular invasion (LVI) on cT_1–2_N_0_M_0_ radiologic solid non-small cell lung cancer (NSCLC) based on a 2-deoxy-2[^18^F]fluoro-D-glucose ([^18^F]F-FDG) positron emission tomography-computed tomography (PET-CT) radiomics analysis.

**Methods:**

The present work retrospectively included 148 patients receiving surgical resection and verified pathologically with cT_1–2_N_0_M_0_ radiologic solid NSCLC. The cases were randomized into training or validation sets in the ratio of 7:3. PET and CT images were used to select optimal radiomics features. Three radiomics predictive models incorporating CT, PET, as well as PET/CT images radiomics features (CT-RS, PET-RS, PET/CT-RS) were developed using logistic analyses. Furthermore, model performance was evaluated by ROC analysis for predicting LVI status. Model performance was evaluated in terms of discrimination, calibration along with clinical utility. Kaplan-Meier curves were employed to analyze the outcome of LVI.

**Results:**

The ROC analysis demonstrated that PET/CT-RS (AUCs were 0.773 and 0.774 for training and validation sets) outperformed both CT-RS(AUCs, 0.727 and 0.752) and PET-RS(AUCs, 0.715 and 0.733). A PET/CT radiology nomogram (PET/CT-model) was developed to estimate LVI; the model demonstrated conspicuous prediction performance for training (C-index, 0.766; 95%CI, 0.728–0.805) and validation sets (C-index, 0.774; 95%CI, 0.702–0.846). Besides, decision curve analysis and calibration curve showed that PET/CT-model provided clinically beneficial effects. Disease-free survival and overall survival varied significantly between LVI and non-LVI cases (P<0.001).

**Conclusions:**

The PET/CT radiomics models could effectively predict LVI on early stage radiologic solid lung cancer and provide support for clinical treatment decisions.

## Introduction

Lung cancer contributes significantly to cancer-associated fatalities worldwide, which caused 18% of total cancer-associated death cases in 2020 (1). Worldwide, non-small cell lung cancer (NSCLC) accounts for 85% of lung cancers (2). As medical treatment develops and the awareness about early diagnosis and treatment of lung disease increases, more cases are detected at an early stage. In patients with early-stage NSCLC, radical surgery and stereotactic ablative body radiotherapy (SABR) remains the key treatment approaches (3–5). Despite providing radical surgical treatment, the relapse rate of early NSCLC is approximately 30–40% (6), with distant metastasis (DM) and regional nodal relapse being the major factors behind treatment failure(65% and 20%, respectively) (7, 8).

Lymphovascular invasion (LVI) refers to cancer cell occurrence in the endothelium-lined lumen or cancer cells-mediated lymphovascular wall destruction. LVI has been reported to occur only in partially solid nodes with a predominantly solid imaging presentation and solid nodes with a solid component of >10 mm (9). In an early-stage NSCLC with a solid imaging presentation, the 5-year recurrence rate increases among cancer cases showing pathological LVI (21.7%) compared with those with no pathological LVI (7.4%), considered a high-risk pathologic feature remarkably increasing relapse and lymph node metastasis incidence (10–13). Patients at high risk for LVI in early-stage NSCLC may benefit from more advanced therapeutic approaches, patients undergoing surgery will require lymph node dissection, which will influence the choice of surgical procedure, while non-surgical patients treated with SABR may require more aggressive adjuvant therapy. Nonetheless, pathology after surgery remains the only tool to confirm LVI, and there is no other validated noninvasive method to predict LVI status preoperatively.

2-deoxy-2[^18^F]fluoro-D-glucose ([^18^F]F-FDG) positron emission tomography-computed tomography (PET-CT) has an important effect on lung cancer stage classification before surgery, relapse, and the assessment of treatment response. Several studies have documented that the fludeoxyglucose (FDG) uptake rate of primary NSCLC lesions correlates with tumor aggressiveness and that some metabolic parameters such as metabolic tumor volume (MTV) and maximum standardized uptake values (SUVmax) are important prognostic factors for lung cancer cases receiving surgical resection. Li C et al. (14) reported that MTV before surgery independently predicted LVI within NSCLC. Noda Y et al. (15)demonstrated that SUVmax of lung cancer could be employed for the identification of LVI. Satoshi et al. (16) reported that LVI and SUVmax were significantly associated with the recurrence of lung cancer. However, further research is warranted to evaluate whether ^18^F-FDG PET/CT could be used to predict LVI before surgery.

Radiomics, a method for high-throughput extraction of features based on medical images, is a useful predictive marker for identifying tumor heterogeneity and other features of the microenvironment invisible to the naked eye (17, 18). Radiomics has proven successful in tumor detection (19), histological and mutational precession (20), prognosis prediction (21), and treatment outcome assessment in lung cancer (22). Nie P et al. (23) constructed a PET/CT-based radiomics nomogram in predicting LVI among 272 lung adenocarcinoma (LAC) cases of stages I-IV, and the results demonstrated the favorable predictive efficacy, however, the group of patients with early-stage solid NSCLC in this study was relatively small, and there is no predictive model for LVI in the solid NSCLC patient population.

Therefore, this study focused on investigating whether PET/CT radiomics nomogram could be applied to predict LVI and its association with patterns of recurrence in cT_1–2_N_0_M_0_ radiologic solid NSCLC patients.

## Materials and methods

### Patients

**Figure 1:**
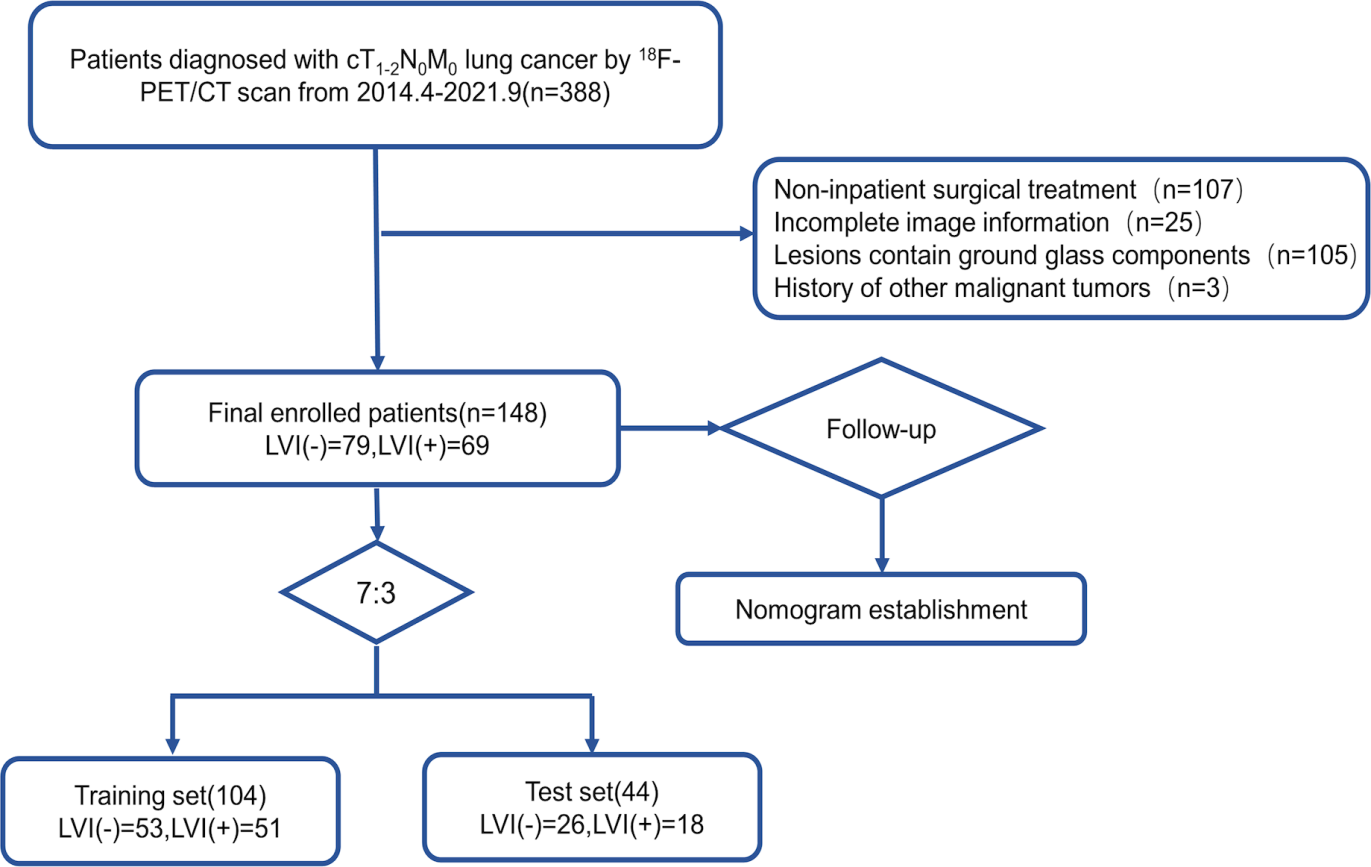
Flow chart illustrating the patient selection and exclusion criteria.

The Institutional Review Board of our institution approved this retrospective study, which waived the requirement for signed informed consent forms. We retrospectively collected 272 lung cancer cases who underwent PET/CT examination at the PET/CT unit in the Affiliated Cancer Hospital of Shandong First Medical University, China, between April 2014 and September 2021; of all the screened cases, 148 fulfilled the criteria. The inclusion criteria were: 1. patients with pathologically confirmed cT_1–2_N_0_M_0_ lung cancer; 2. patients who underwent PET/CT scan before surgery; 3. patients in whom PET/CT showed positive FDG uptake at the primary tumor site. Exclusion criteria were: 1. CT in patients demonstrated lesions showing ground glass components; 2. patients had received preoperative antitumor therapy; 3. Patients did not undergo surgery at our institution earlier;4. patients with other malignancies; 5. patients with other lung diseases that could affect image analysis. **Figure 1** illustrates the screening process of patients who were stratified into training and validation groups (7:3 ratio). Training set and validation set samples were applied in predicting the training model and in evaluating model performance, respectively.

All cases were retrieved to collect participants’ baseline clinical information, including their age, gender, smoking history, clinical T-stage, AJCC cancer staging system-based TNM stage, primary foci SUVmax, SUVmean, MTV, total lesion glycolysis (TLG), and tumor location by deriving PET/CT image information. An expert pathologist with ten years of clinical experience interpreted the pathology, including pathological type, pleural invasion, and LVI statuses of the patients.

### PET/CT image acquisition

All patients underwent 6-h fasting before obtaining ^18^F-FDG PET/CT images. Blood glucose (BG) contents of the patients were <11.0 mmol/L measured prior to the scan. All patients underwent scanning using Gemini TF Big Bore PET/CT system (Philips Medical Systems); the PET tracer was produced using the MINI Trace (GE Tracer Lab, USA), which produces its own labeled nuclide ^18^F-FDG was automatically synthesized with a purity of ≥5%. FDG (3.7–5.5 MBq/kg) was injected intravenously in the patients who rested calmly for 60 min; the bladder was emptied before image acquisition, and the spiral CT scan was provided 120 kV, 50 mA tube current, as well as 4 mm layer thickness, and then PET scan with 4 mm layer thickness, corrected for CT attenuation and ordered subsets. Through adopting ordered subsets maximization and CT attenuation correction, patient reconstruction was implemented. Besides, all patients underwent end-inspiratory breath-hold spiral chest CT scans (layer thickness, 5 mm).

### Image analysis

Two Nuclear Medicine physicians who were blind to pathological or clinical data analyzed the PET/CT images and consistently analyzed the following features: “According to the thin-layer CT presentation, the solid component is the part of the nodule without identifiable vascular and bronchial structures.” The current study deemed a “solid” tumor to be the maximal solid tumor diameter to maximal tumor diameter ratio (called consolidation-to-tumor ratio, CTR >0.5 (24, 25). SUVmax to be the maximum value on the highest counted pixel within one region of interest (ROI) containing the whole tumor by plotting it onto an axial PET image. MTV was extracted by the target area outlining software 3D-Slicer through an iterative adaptive algorithm to compute the threshold value for outlining the tumor edge. TLG = SUVmean*MTV (cm^3^).

### Tumor segmentation and radiomics feature extraction

**Figure 2:**
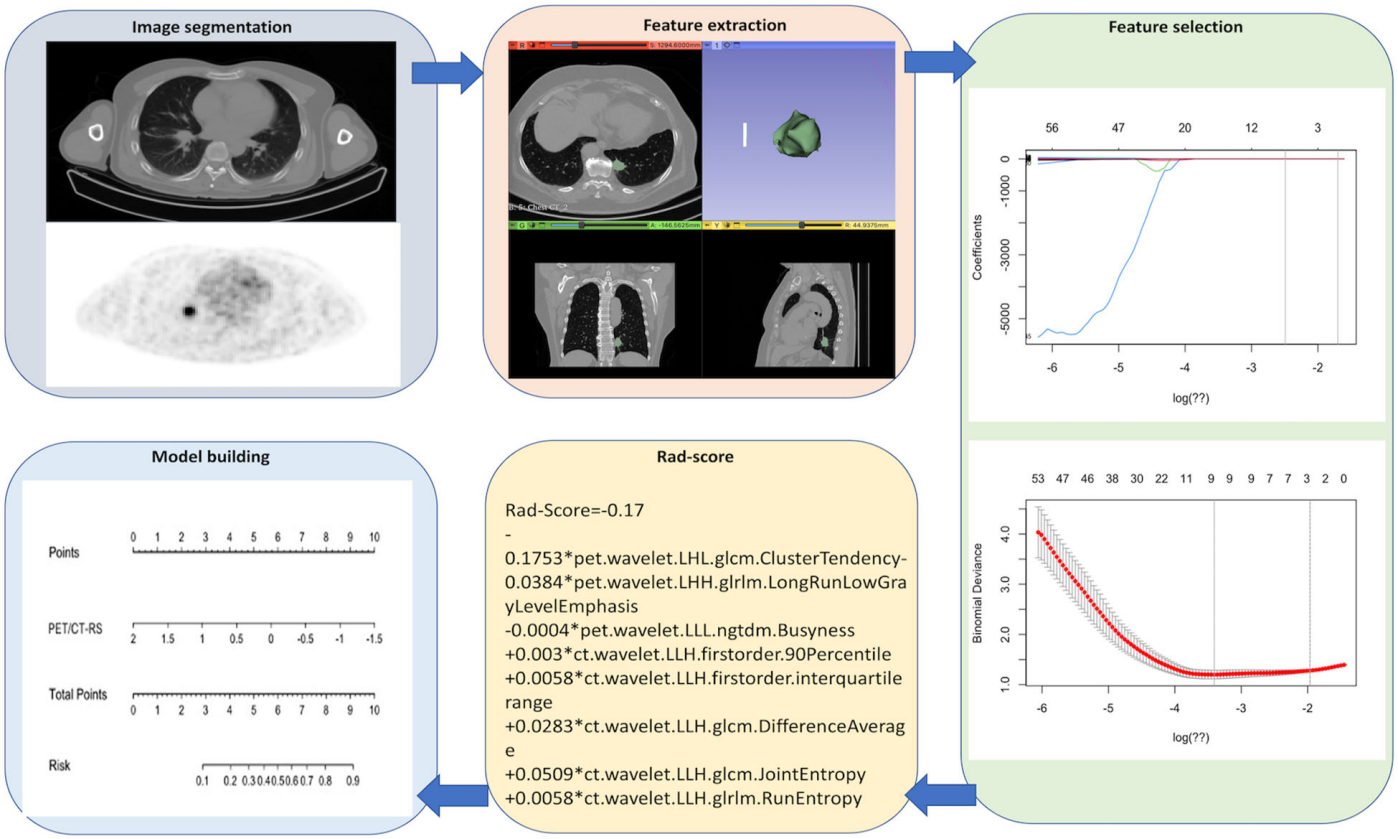
The flow diagram of this study. "*" means multiply.

**Figure 2** shows the radiomics collection flowchart. Digital Imaging and Communications (DICOM) standard format images of the patient’s ^18^F-FDG PET/CT were imported into the 3D-Slicer software tool (version 4.13.0, 1.0, www.slicer.org) to segment the tumor. Thereafter, the tumor lesions were outlined on the axial PET as well as CT images, and the CT images were manually outlined layer-wise by the radiologist. Initially, the PET images were automatically outlined by a fixed threshold of SUVmax>2.5 and subsequently manually corrected by the radiologist. In total, 1702 radiomics features (851 for CT and 851 for PET) were obtained from the volume-of-interest (VOI), including 162 first-order features, 14 shape features, 264 gray-level co-occurrence matrix (GLCM) features, 144 gray-level size region matrix (GLSZM) features, 144 gray-level run length matrix (GLRLM) features, 45 neighborhood gray level difference matrix (NGTDM) features, and 126 gray level dependence matrix (GLDM) features.

The intra- and inter-class correlation coefficients (ICCs) were used to evaluate feature collection reproducibility in and between the two readers. Readers 1 and 2 randomly selected 20 (10 with LVI, 10 without LVI) PET and CT images in the entire set. Reader 1 repeated this segmentation process after two weeks. The ICC >0.80 indicates high consistency in feature collection. Reader 1 was responsible for segmenting the ROI of all the remaining patients.

### Feature selection and radiomics signature establishment

To avoid overfitting, the features were downscaled according to the following three steps before feature construction. First, radiomics features whose intra- and inter-reader ICCs >0.80 were retained to avoid any subjective heterogeneity of ROI segmentation. Second, a one-way analysis of variance (ANOVA) was performed to select features of p<0.05 between the patients with LVI and without LVI. Finally, optimal radiomics features were selected using the least absolute shrinkage and selection operator algorithm (LASSO) to construct a radiomics signature (RS). Thereafter, patients’ Rad-scores were determined.

### Predictive model development and validation

The current study developed the CT-RS, PET-RS, and PET/CT-RS machine learning models to predict LVI in cT_1–2_N_0_M_0_ lung cancer. Values of sensitivity, specificity, and area under the ROC curve (AUC) were determined to assess their diagnostic accuracy. Then, an optimal model for generating a radiomics nomogram was selected. Model clinical utility was studied using decision curve analysis (DCA) as well as calibration curve analysis.

### Follow-up and survival

CT examination was conducted during the follow-up period at 6–12-month intervals within the initial 2 years after surgery but at 1-year intervals within 5 years. Our study endpoints included overall survival (OS) and Disease-free survival (DFS); OS was deemed as the duration between surgery and death, and DFS as the duration between relapse and disease progression. Kaplan-Meier approach was used for plotting survival curves.

### Statistical analyses

IBM SPSS version 26.0 (https://www.ibm.com) was employed to select key statistical variables through univariate (Mann-Whitney U test and chi-square test) as well as multivariate regression. R software (version 4.1.3, https://www.r-project.org) was adopted for ICC, DCA, calibration plots, and survival analysis. The MedCalc statistical software (version 19.0.7, https://www.medcalc.org) was used to analyze the ROC curve. A p-value of <0.05 (two-sided) indicated statistical significance.

## Results

### Patient characteristics

In this study, a total of 148 patients (94 M and 54 F, aged 31–85 (median, 64) years) were recruited, including 69(47%) patients with pathological LVI and 79 (53%) patients without pathological LVI. **Table 1** shows clinical factors, pathological information, and PET metabolic factors. Patients with LVI had higher values of SUVmax, SUVmean, MTV, and TLG than those without LVI. Gender and tumor location were found statistically significant (*P*<0.05). The distribution of age, smoking history, type of pathology, pleural invasion, and AJCC stage showed similarity in both groups (*P*>0.05), but there was no statistically significant difference (*P*>0.05) upon multivariate regression (**Table 2**).

**Table 1:** Baseline clinical characteristics of the patients in the training and validation sets.

Clinical factors	Training set (n=104)	Validation set (n=44)	P
**Gender**			0.254
Female	41 (39)	13 (30)	
Male	63 (61)	31 (70)	
**Smoking history**			0.740
Negative	56 (54)	25 (57)	
Positive	48 (46)	19 (43)	
**Pathological types**			0.326
LAC	80 (77)	30 (68)	
SCC	24 (23)	14 (32)	
**Pleural invasion**			0.808
Negative	73 (70)	30 (68)	
Positive	31 (30)	14 (32)	
**T stage**			0.052
T1a	0 (0)	1 (1)	
T1b	30 (29)	9 (20)	
T1c	46 (44)	13 (30)	
T2a	21 (20)	14 (32)	
T2b	7 (7)	7 (16)	
**Tumor location**			0.729
Central	21 (20)	10 (23)	
Peripheral	83 (80)	24 (77)	
**Age**	61.00 (54.00-70.75)	66.00 (60.00-73.00)	0.090
**SUVmax**	7.27 (5.08-10.78)	9.92 (5.66-12.33)	0.081
**SUVmean**	3.88 (3.28-4.72)	4.15 (3.50-5.42)	0.127
**MTV**	6.60 (2.73-13.58)	11.38 (5.06-19.75)	0.089
**TLG**	30.96 (8.46-59.64)	41.78 (18.22-88.61)	0.123

SUVmax, maximum standardized uptake value; SUVmean, mean standardized uptake value; MTV, metabolic tumor volume; TLG, total lesion glycolysis; LVI, lymphovascular invasion; SCC, squamous cell carcinoma; LAC, lung adenocarcinoma.

**Table 2:** Univariate and Multivariate analysis to significant for LVI.

Characteristic	Univariable Analysis P value	Multivariable analysis		
		P value	HR	95%CI
**Gender(**Male vs Female**)**	0.014	0.076	0.454	0.189-1.087
**Smoking history** **(**Negative vs Positive)	0.079	–	–	–
**Pathological types** (LAC vs SCC)	0.424	–	–	–
**Pleural invasion** **(**Negative vs Positive)	0.931	–	–	–
**T stage**	0.239	–	–	–
T1b vs T1c	0.072			
T1b vs T2a	0.091			
T1b vs T2b	0.242			
**Tumor location** **(**Central vs Peripheral)	0.022	0.156	0.422	0.129-1.388
**Age**	0.243	–	–	–
**SUVmax**	0.003	0.251	0.790	0.529-1.18
**SUVmean**	0.003	0.167	3.136	0.620-15.86
**MTV**	0.001	0.644	0.967	0.840-1.11
**TLG**	<0.001	0.520	1.011	0.978-1.04

SUVmax, maximum standardized uptake value; SUVmean, mean standardized uptake value; MTV, metabolic tumor volume; TLG, total lesion glycolysis; LVI, lymphovascular invasion; SCC, squamous cell carcinoma; LAC, lung adenocarcinoma. "-" means they were not included in the multi-factor analysis.

### Construction and validation of radiomics model

From the 1702 radiomics features collected in 3D ROI on PET and CT images, those with ICC value <0.80 (numbering 457) were eliminated, and 551 PET and 694 CT features were retained. Overall, 40 PET and 238 CT features were found to be insignificant in LVI compared with non-LVI patients (P = 0.00–0.05) and were incorporated in LASSO analysis. Finally, we chose 5 CT and 3 PET features to construct CT-RS, PET-RS, and PET/CT-RS, respectively, for predicting LVI status within cT_1–2_N_0_M_0_ lung cancer. The **Supplementary Table** demonstrates radiomics features as well as the associated Rad-score formulas and coefficients.

**Figure 3:**
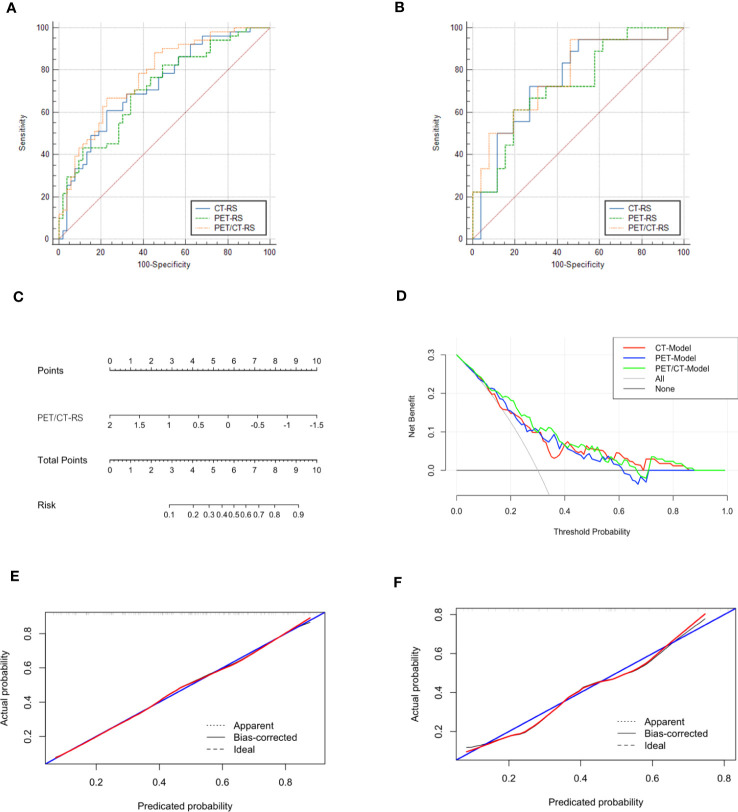
**(A)** ROCs of different radiomics models in the training set, The three colors represent the three models, blue: CT-RS model, green: PET-RS model, yellow: PET/CT-RS model; **(B)** ROCs of different radiomics models in the validation set; **(C)** The radiomics nomogram: PET/CT RadScore-Model (PET/CT-Model); **(D)** Decision curve analysis for CT-Model, PET-Model, and PET/CT-Model; **(E)** Calibration curve of the PET/CT Model in the training set; **(F)** Calibration curve of the PET/CT Model in the validation set.

Differences in PET/CT-RS features between the LVI and non-LVI patients were found statistically significant (*P*<0.05) for both sets. The maximum AUC value was attained by PET/CT-RS (AUC values for training and validation, 0.773, 0.774, respectively), and both datasets achieved remarkable sensitivity and specificity (sensitivity = 0.774, 0.538; specificity = 0.667, 0.944, training and validation, respectively). **Figure 3** depicts the ROC curves. CT-RS and PET-RS had AUC values of 0.727 and 0.715, respectively, for the training set, whereas values of 0.752 and 0.733, respectively, for the validation set. Sensitivities and specificities achieved by the CT-RS model were 0.774 and 0.608, respectively, while those by the PET-RS model were 0.642 and 0.652, respectively (**Table 3**).

**Table 3:** Different radiomics models predict the diagnostic performance of LVI.

	CT-RS		PET-RS		PET/CT-RS	
	Training set	validation set	Training set	validation set	Training set	validation set
**AUC**	0.727	0.752	0.715	0.733	0.773	0.774
**Sensitivity**	0.774	0.731	0.642	0.815	0.774	0.538
**Specificity**	0.608	0.722	0.652	0.611	0.667	0.944

PET/CT-RS showed the best efficacy in predicting LVI, and the C-index of radiomics nomogram (PET/CT-Model) was developed based on PET/CT-RS with 0.766 (95% CI: 0.728–0.805, training set) and 0.774 (95% CI: 0.702–0.846, validation set). **Figure 3** depicts the diagnostic performance, calibration curves, and decision curves of the model. The calibration curve revealed that the estimated risks conformed to the observed LVI outcomes. Besides, the diagonal curved line approximated the ideal straight line, suggesting the accuracy of our PET/CT-Model-based nomogram in prognosis prediction. Furthermore, DCA revealed the benefits of the PET/CT model in predicting LVI.

### Survival outcomes

**Figure 4:**
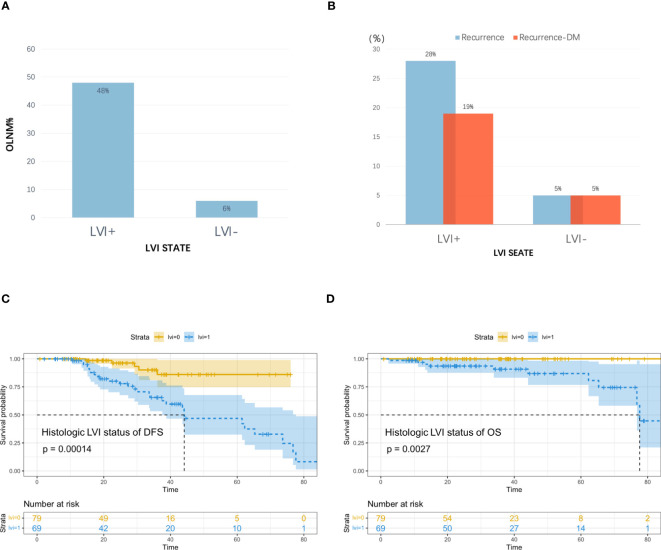
**(A)** Incidence of occult lymph node metastasis (OLNM), **(B)** Recurrence rate and distant metastasis rate in patients with and without pathological LVI; **(C)** Disease Free Survival (DFS) curves according to pathologic LVI status, **(D)** Overall Survival (OS) curves according to pathologic LVI status.

By April 10, 2022, we had successfully followed up on 148 patients (median, 34.33 months; 95% CI, 27.88–40.79). The overall recurrence rate was 22% (33/148, including 41% (28/69) of patients with LVI and 6% (5/79) of patients without LVI), and DM occurred in 16% of the patients (24/148, including 28% (19/69) of patients with LVI and 6% (5/79) without LVI); median DFS for overall patients was 73.67 (range 1–85) and 44.13 (95% CI, 37.57–77.60) months for patients with LVI. The overall mortality rate was 7% (10/148), and all patients had LVI status. **Figure 4** shows the relapse rate, mortality, and survival curves.

Analyses of the recurrence pattern of 148 cT_1–2_N_0_M_0_ lung cancer patients treated with radical surgery showed that 26% of cases had occult lymph node metastases (OLNM), including 6% (5/79) of cases without LVI and 48% (33/69) with LVI, and the incidence of OLNM among LVI cases showed marked increase and they were at greater risk of lymph node metastases (LNM). The overall cohort had a recurrence rate of 22% after radical surgery, with DM being the most common cancer type. The recurrence rate increased significantly among patients with LVI compared with those without LVI; therefore, because of the worse prognosis for survival, more aggressive adjuvant therapy may be needed for this group of high-risk patients with LVI to ensure radical treatment, improve tumor control rates, and improve patient recurrence-free survival and overall survival.

## Discussion

The current study focused on investigating the significance of PET/CT radiomics in predicting LVI status and prognosis before surgery in cases developing early radiologic solid lung cancer. It was found that PET/CT radiomics nomogram achieved good LVI predictive ability among cases developing cT_1–2_N_0_M_0_ radiologic solid lung cancer. Besides, the incidence of OLNM and postoperative recurrence rates were found to be remarkably higher in patients with LVI, suggesting a greater risk of LNM as well as a worse prognosis. OS and DFS differed significantly between cases with LVI and without LVI, highlighting the importance of LVI as an aid to clinical treatment decisions in patients with radiologic solid lung cancer at an early stage.

^18^F-FDG PET/CT plays an important role in evaluating the clinical staging as well as the prognosis of lung cancer. In our study, we observed that SUVmax and SUVmean, MTV, and TLG were statistically different in patients with LVI and without LVI (*P*<0.05). Partially corroborating the earlier findings by C Li et al., C Li et al. (14) examined PET/CT image parameters along with clinical features in 161 cases with NSCLC and documented that tumor MTV independently predicted LVI (p<0.05). In addition, we found that no variable was considered to be an independent risk factor for LVI, probably because the training set had fewer samples. It indicates that conventional features collected on traditional images contributed little to LVI. Therefore, it is suggested to develop further credible and objective biomarkers for identifying LVI status among lung cancer cases.

High-throughput collection for high-level quantitative features can be employed in radiomics to characterize tumor phenotypes objectively and quantitatively; they can be collected in medical studies by adopting advanced mathematical algorithms for imaging data identifying tumor biological and histological features, besides visually assessing CT, MRI, and PET/CT images. The diagnostic efficacy of radiomics has previously been demonstrated in preoperatively predicting LVI in different cancers. Zhang Y et al. (26) constructed the multimodal radiomics model (MR/CT) to predict LVI in 94 rectal cancer cases; this model was validated based on the training set (AUC, 0.884; 95% CI 0.803- 0.964) as well as the validation set (AUC, 0.876; 95% CI 0.721–1.000) and demonstrated remarkable predictive power. In a radiomics study on predicting LVI status before surgery in gastric cancer patients, Chen et al. (27) proposed that a combined model based on radiomics features of enhanced CT combined with clinical factors could effectively predict LVI status before surgery in gastric cancer patients (AUC, 0.856) of training cohort; Yang et al. (28) stated that AUCs of PET/CT-based radiomics models reached 0.881 and 0.854 (training and validation groups). In constructing a radiomics model for 272 patients with LAD, Nie P et al. (23) documented that AUC was not significantly different in CT compared with PET radiomics models, and the PET/CT-based radiomics model achieved an increased net benefit compared with PET and CT models. In partial agreement with the earlier findings of Nie P et al., we observed that the AUC of PET/CT-RS was better than that of both CT-RS and PET-RS. Our constructed PET/CT model proved feasible in predicting LVI. However, at the same time, our results found that the sensitivity of PET/CT models in the validation group was low, which was related to the significant improvement of model specificity in the validation group, and the improvement of model specificity was accompanied by the reduction of sensitivity. Further improvement of model performance may reduce the gap between the two. The reason for this result may due to the relatively small amount of data in the validation set and the bias of some missing values when performing model validation verification, but the overall performance of the PET/CT model is still optimal. In the follow-up study, we will try to increase the sample size as much as possible.

Currently, LVI is not included in the AJCC guidelines to independently predict prognosis in the TNM classification system for lung cancer. However, LVI has been identified as a factor predicting the prognosis of surgically treated patients with lung cancer, which is associated with tumor recurrence (16). In the present study, the OLNM rate significantly increased among LVI cases, the DFS and OS of patients with LVI were significantly worse than that of patients without LVI. A potential explanation for the results may be as follows: patients with LVI showed more aggressive disease than those without LVI. Presently, for patients with early-stage lung cancer without obvious contraindications to surgery, surgery is clinically recommended as the preferred treatment intervention. For patients who are older, have poor lung function and cardiac insufficiency, and cannot tolerate surgical operations, other treatment modalities, such as radical radiotherapy, can be employed. Previous studies have shown that suitable radical radiotherapy can achieve results similar to those in surgery (29). In patients with early lung cancer who cannot tolerate surgery, precise pathological lymph node staging is not available, which may underestimate the risk of recurrence and delay treatment of the disease. PET/CT radiomics may offer good predictive value for LVI in patients with early-stage inoperable radiologic solid lung cancer.

However, this study has a few limitations. First, the included cases were limited in number, more samples are needed to further validate our results and assumptions and analyze model stability. Second, Multi-center research perhaps provide more data to build more representative models, so the inclusion of external validation would have been better. Given that radiomics involves extracting information from images when conducting multi-center research, we need to review the machine parameters and operating procedures of the collected images to exclude errors caused by human or machine differences. Finally, because CT tumor segmentation is done manually, the development of a more efficient tumor segmentation method remains a key consideration.

## Conclusions

In the current work, radiomics features extracted from PET/CT imaging can be adopted for efficiently predicting LVI status on cT_1–2_N_0_M_0_ radiologic solid lung cancer, which can improve patient diagnosis before surgery and make personalized treatment planning.

## Data availability statement

The raw data supporting the conclusions of this article will be made available by the authors, without undue reservation.

## Ethics statement

This retrospective study was approved by the Institutional Review Board (IRB) of Shandong Cancer Hospital, which waived the requirement for signed informed consent. Written informed consent for participation was not required for this study in accordance with the national legislation and the institutional requirements.

## Author contributions

Among the authors in the list, JW and XS has done a lot of work in research design, data collection, paper writing modification, and paper finalization. JW and JL participated in the data collection. ZZ made some contributions to the data analysis and writing of the article. XS and LX has done a lot of work in research design and paper finalization. The first draft of the manuscript was written by JW and XS. All authors contributed to the article and approved the submitted version.
