# Supplementary material for: 18F-FDG PET/CT radiomics for prediction of lymphovascular invasion in patients with early stage non-small cell lung cancer
Source: Front Oncol. 2023 Jul 21;13:1185808. doi: 10.3389/fonc.2023.1185808 (PMC10401837; doi:10.3389/fonc.2023.1185808)
Supplement: Supplementary file 1 [file DataSheet_1.docx]

***Supplementary Material***

**^18^F-FDG PET/CT Radiomics for Prediction of Lymphovascular Invasion in Patients with Early Stage Non-Small Cell Lung Cancer**

Jie Wang, Zhonghang Zheng, Yi Zhang, Weiyue Tan, Jing Li, Ligang Xing, Xiaorong Sun*

***Correspondence:** Xiaorong Sun, MD, PhD, Department of Nuclear Medicine, Shandong Cancer Hospital and Institute, Shandong First Medical University and Shandong Academy of Medical Sciences, 440 Jiyan Road, Jinan, Shandong 250117, China.

Email: [251400067@qq.com](mailto:251400067@qq.com)

Tel/Fax:86-531-67626287

ORCID：0000-0001-8520-9556

**Supplementary Table** Radiomics features for calculating PET/CT radiomics score (Rad-scores) of LVI and their importance

| **Feature name** |  | **Importance** | |
| --- | --- | --- | --- |
| pet.wavelet.LHL.glcm.ClusterTendency |  | -0.1753 |  |
| pet.wavelet.LHH.glrlm.LongRunLowGrayLevelEmphasis |  | -0.0384 |  |
| pet.wavelet.LLL.ngtdm.Busyness |  | -0.0004 |  |
| ct.wavelet.LLH.firstorder.90Percentile |  | 0.003 |  |
| ct.wavelet.LLH.firstorder.InterquartileRange |  | 0.0058 |  |
| ct.wavelet.LLH.glcm.DifferenceAverage |  | 0.0283 |  |
| ct.wavelet.LLH.glcm.JointEntropy |  | 0.0509 |  |
| ct.wavelet.LLH.glrlm.RunEntropy |  | 0.0058 |  |

The rad-score calculation formula is reported below, wherein the sum of the value of every element is multiplied by its coefficient.

*Rad − score =-0.17*

*-0.1753*pet.wavelet.LHL.glcm.ClusterTendency*

*-0.0384*pet.wavelet.LHH.glrlm.LongRunLowGrayLevelEmphasis*

*-0.0004*pet.wavelet.LLL.ngtdm.Busyness*

*+0.003*ct.wavelet.LLH.firstorder.90Percentile*

*+0.0058*ct.wavelet.LLH.firstorder.InterquartileRange*

*+0.0283*ct.wavelet.LLH.glcm.DifferenceAverage*

*+0.0509*ct.wavelet.LLH.glcm.JointEntropy*

*+0.0058*ct.wavelet.LLH.glrlm.RunEntropy*

The definition and quantization methods of radiomics features are shown as follows, which were cited from Pyradiomics document (Version 2.1.2., Page 35-65, https://

pyradiomics.readthedocs.io).

**1. [pet.wavelet.LHL.glcm.ClusterTendency]**

Cluster Tendency


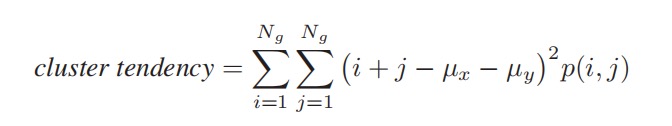


Cluster Tendency is a measure of groupings of voxels with similar gray-level values.

**2. [pet.wavelet.LHH.glrlm.LongRunLowGrayLevelEmphasis]**

Long Run Low Gray Level Emphasis (LRLGLE)


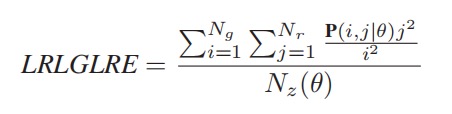


LRLGLRE measures the joint distribution of long run lengths with lower gray-level values.

**3. [pet.wavelet.LLL.ngtdm.Busyness]**

**
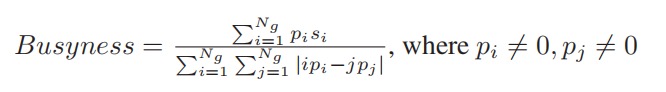
**

A measure of the change from a pixel to its neighbour. A high value for busyness indicates a ‘busy’ image, with rapid changes of intensity between pixels and its neighbourhood.

**4. [ct.wavelet.LLH.firstorder.90Percentile]**

90th percentile

The 90th percentile of **X**

**5. [ct.wavelet.LLH.firstorder.InterquartileRange]**

Interquartile Range


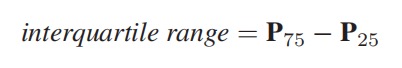


Here P25 and P75 are the 25th and 75th percentile of the image array, respectively.

**6. [ct.wavelet.LLH.glcm.DifferenceAverage]**

Difference Average


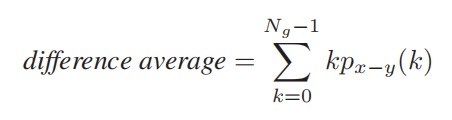


Difference Average measures the relationship between occurrences of pairs with similar intensity values and occurrences of pairs with differing intensity values.

**7. [ct.wavelet.LLH.glcm.JointEntropy]**

Joint Entropy


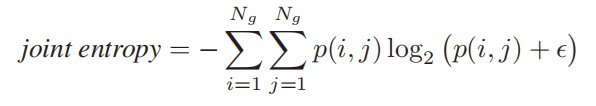


Joint entropy is a measure of the randomness/variability in neighborhood intensity values.

**8. [ct.wavelet.LLH.glrlm.RunEntropy]**

Run Entropy (RE)


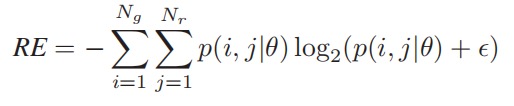


Here, *∊* is an arbitrarily small positive number (*≈* 2*.*2 *×* 10*^−^*^16^).

RE measures the uncertainty/randomness in the distribution of run lengths and gray levels. A higher value indicates more heterogeneity in the texture patterns.
